# Extracellular vesicles secreted by human aneuploid embryos present a distinct transcriptomic profile and upregulate MUC1 transcription in decidualised endometrial stromal cells

**DOI:** 10.1093/hropen/hoae014

**Published:** 2024-03-12

**Authors:** Sofia Makieva, Elisa Giacomini, Giulia Maria Scotti, Dejan Lazarevic, Valentina Pavone, Jessica Ottolina, Ludovica Bartiromo, Matteo Schimberni, Marco Morelli, Alessandra Alteri, Sabrina Minetto, Giovanni Tonon, Massimo Candiani, Enrico Papaleo, Paola Viganò

**Affiliations:** Reproductive Sciences Laboratory, IRCCS San Raffaele Scientific Institute, Milan, Italy; Reproductive Sciences Laboratory, IRCCS San Raffaele Scientific Institute, Milan, Italy; Centre for Omics Sciences, IRCCS San Raffaele Scientific Institute, Milan, Italy; Centre for Omics Sciences, IRCCS San Raffaele Scientific Institute, Milan, Italy; Reproductive Sciences Laboratory, IRCCS San Raffaele Scientific Institute, Milan, Italy; Centro Scienze della Natalità, IRCCS San Raffaele Scientific Institute, Milan, Italy; Department of Obstetrics and Gynaecology, IRCCS Scientific Institute San Raffaele, Milan, Italy; Department of Obstetrics and Gynaecology, IRCCS Scientific Institute San Raffaele, Milan, Italy; Centre for Omics Sciences, IRCCS San Raffaele Scientific Institute, Milan, Italy; Centro Scienze della Natalità, IRCCS San Raffaele Scientific Institute, Milan, Italy; Centro Scienze della Natalità, IRCCS San Raffaele Scientific Institute, Milan, Italy; Centre for Omics Sciences, IRCCS San Raffaele Scientific Institute, Milan, Italy; Department of Obstetrics and Gynaecology, IRCCS Scientific Institute San Raffaele, Milan, Italy; Centro Scienze della Natalità, IRCCS San Raffaele Scientific Institute, Milan, Italy; Reproductive Sciences Laboratory, IRCCS San Raffaele Scientific Institute, Milan, Italy

**Keywords:** extracellular vesicles, embryo, aneuploidy, non-invasive preimplantation genetic testing for aneuploidy, implantation, endometrium

## Abstract

**STUDY QUESTION:**

Do extracellular vesicles (EVs) secreted by aneuploid human embryos possess a unique transcriptomic profile that elicits a relevant transcriptomic response in decidualized primary endometrial stromal cells (dESCs)?

**SUMMARY ANSWER:**

Aneuploid embryo-derived EVs contain transcripts of *PPM1J*, *LINC00561*, *ANKRD34C*, and *TMED10* with differential abundance from euploid embryo-derived EVs and induce upregulation of *MUC1* transcript in dESCs.

**WHAT IS KNOWN ALREADY:**

We have previously reported that IVF embryos secrete EVs that can be internalized by ESCs, conceptualizing that successful implantation to the endometrium is facilitated by EVs. Whether these EVs may additionally serve as biomarkers of ploidy status is unknown.

**STUDY DESIGN, SIZE, DURATION:**

Embryos destined for biopsy for preimplantation genetic testing for aneuploidy (PGT-A) were grown under standard conditions. Spent media (30 μl) were collected from euploid (n = 175) and aneuploid (n = 140) embryos at cleavage (Days 1–3) stage and from euploid (n = 187) and aneuploid (n = 142) embryos at blastocyst (Days 3–5) stage. Media samples from n = 35 cleavage-stage embryos were pooled in order to obtain five euploid and four aneuploid pools. Similarly, media samples from blastocysts were pooled to create one euploid and one aneuploid pool. ESCs were obtained from five women undergoing diagnostic laparoscopy.

**PARTICIPANTS/MATERIALS, SETTING, METHODS:**

EVs were isolated from pools of media by differential centrifugation and EV-RNA sequencing was performed following a single-cell approach that circumvents RNA extraction. ESCs were decidualized (estradiol: 10 nM, progesterone: 1 µM, cAMP: 0.5 mM twice every 48 h) and incubated for 24 h with EVs (50 ng/ml). RNA sequencing was performed on ESCs.

**MAIN RESULTS AND THE ROLE OF CHANCE:**

Aneuploid cleavage stage embryos secreted EVs that were less abundant in RNA fragments originating from the genes *PPM1J* (log2fc = −5.13, *P* = 0.011), *LINC00561* (log2fc = −7.87, *P* = 0.010), and *ANKRD34C* (log2fc = −7.30, *P* = 0.017) and more abundant in *TMED10* (log2fc = 1.63, *P* = 0.025) compared to EVs of euploid embryos. Decidualization *per se* induced downregulation of *MUC1* (log2fc = −0.54, *P* = 0.0028) in ESCs as a prerequisite for the establishment of receptive endometrium. The expression of *MUC1* transcript in decidualized ESCs was significantly increased following treatment with aneuploid compared to euploid embryo-secreted EVs (log2fc = 0.85, *P* = 0.0201).

**LARGE SCALE DATA:**

Raw data have been uploaded to GEO (accession number GSE234338).

**LIMITATIONS, REASONS FOR CAUTION:**

The findings of the study will require validation utilizing a second cohort of EV samples.

**WIDER IMPLICATIONS OF THE FINDINGS:**

The discovery that the transcriptomic profile of EVs secreted from aneuploid cleavage stage embryos differs from that of euploid embryos supports the possibility to develop a non-invasive methodology for PGT-A. The upregulation of *MUC1* in dESCs following aneuploid embryo EV treatment proposes a new mechanism underlying implantation failure.

**STUDY FUNDING/COMPETING INTEREST(S):**

The study was supported by a Marie Skłodowska-Curie Actions fellowship awarded to SM by the European Commission (CERVINO grant agreement ID: 79620) and by a BIRTH research grant from Theramex HQ UK Ltd. The authors have no conflicts of interest to declare.

WHAT DOES THIS MEAN FOR PATIENTS? Preimplantation genetic testing for aneuploidy (PGT-A) is increasingly practiced to select a healthy embryo for transfer after *in vitro* fertilization (IVF) treatment. Although there is an undoubted rationale behind the theory of transferring a healthy embryo, the practice as such does not seem to increase the chance of pregnancy in all cases. It may be that the invasive nature of embryo biopsy, a methodology required for PGT-A, is compromising the ability of healthy embryos to implant. What is more, the use of embryo biopsy is highly debatable owing to its lack of proven long-term biosafety. Developing non-invasive methodologies for PGT-A, for example, a ‘liquid biopsy’ whereby the embryo is diagnosed as healthy or unhealthy based on the analysis of the liquid in which it was cultured, could replace biopsy. To develop such strategies, we require a better understanding of the routes that healthy embryos employ to implant in the womb. Our study shows that unhealthy embryos secrete into the culture liquid small particles (called vesicles) loaded with specific molecules. These particles and their molecules could be internalized by the cells populating the womb to regulate the decision of the cells to reject the embryo. Our work reveals that exposure of womb cells to such unhealthy embryonic particles induces an increase in an important protein that might block implantation. These findings could be extrapolated to inform the development of non-invasive PGT-A and to better understand the mechanisms of rejection of unhealthy embryos.

## Introduction

Extracellular vesicles (EVs) are small, membrane-bound particles released by cells and can be found in various bodily fluids, including spent media from embryos (Giacomini *et al.*, 2021). Recent findings suggest a potential role of embryo-secreted EVs in the establishment of embryo implantation through both autocrine and paracrine mechanisms (Capra and Lange-Consiglio, 2020; Aleksejeva *et al.*, 2022).

A few years ago, our group characterized EVs secreted by human ICSI-created embryos and demonstrated that they can be internalized by endometrial cells (Giacomini *et al.*, 2017). Others have shown that bovine embryos that develop to the blastocyst stage secrete EVs enriched in a distinct miRNA, which, when administered in a synthetic form, could significantly increase the capacity of blastocysts to hatch and consequently implant (Pavani *et al.*, 2022). The transfer of RNA transcripts from EVs secreted by human trophoblast spheroids to endometrial cells has been demonstrated. As a result, the expression of endometrial genes critical for implantation would be modified (Es-Haghi *et al.*, 2019).

Beyond their role in embryo–endometrial dialog, interest in embryo-derived EVs derives from their possible usefulness as biomarkers of embryo ploidy status. Nowadays, preimplantation genetic testing for aneuploidy (PGT-A), used to identify chromosomal anomalies in *in vitro* fertilized embryos, is performed by an invasive biopsy, which involves removing a small number of cells from the developing embryo for genetic analysis (Yan *et al.*, 2021; Cheng *et al.*, 2022; Kucherov *et al.*, 2023). Safety concerns have been raised, albeit not consistently (Tiegs *et al.*, 2021), in relation to the procedure of the embryo biopsy itself, which may compromise embryo competence, leading to the inability of euploid blastocysts to implant (Pagliardini *et al.*, 2020; Cimadomo *et al.*, 2023). Thus, non-invasive approaches have been explored as potential alternatives that may help improve the practice of embryo selection. For example, screening spent culture medium and analysis of blastocoelic fluid have shown promise for detecting chromosomal abnormalities in embryos without the need for biopsy (Vera-Rodriguez *et al.*, 2018; Gianaroli *et al.*, 2023; Griffin *et al.*, 2023). This type of screening involves analysis of the media or blastocoelic fluid for the presence of ploidy biomarkers that could represent the embryo as a whole (Belandres *et al.*, 2019_;_Griffin *et al.*, 2023).

Although embryo-secreted EVs appear to be important in the establishment of implantation, no study to date has examined whether euploid and aneuploid human embryos secrete EVs with a diverse transcriptomic profile that can disclose their genetic makeup, and if aneuploid embryos secrete EVs that can induce a transcriptomic response that is incompatible with implantation in the decidualized endometrium. We set out to explore these research questions.

## Materials and methods

### Human samples

This study was approved by the Institutional Ethical Committee (CERVINO-796206; Comitato etico IRCCS Ospedale San Raffaele) and all subjects involved provided written informed consent. Endometrial biopsies were collected from women (n = 5) undergoing laparoscopy for ovarian benign pathology, using a surgical curette, at the Obstetrics and Gynaecology clinic. All women were younger than 40 years and had regular menstrual cycles. Women with uterine disorders or who had received oral contraceptives, progestins, GnRH analogs/antagonists, or other hormonal medications in the last 3 months were excluded from the study.

The embryo spent culture medium samples were obtained from patients attending the Centro Science Natalità of the San Raffaele Hospital in Milano, with an indication to ART treatment and with or without indication to PGT-A. All the experiments were performed in accordance to the principles set out in the World Medical Association Declaration of Helsinki.

**Figure 1.**
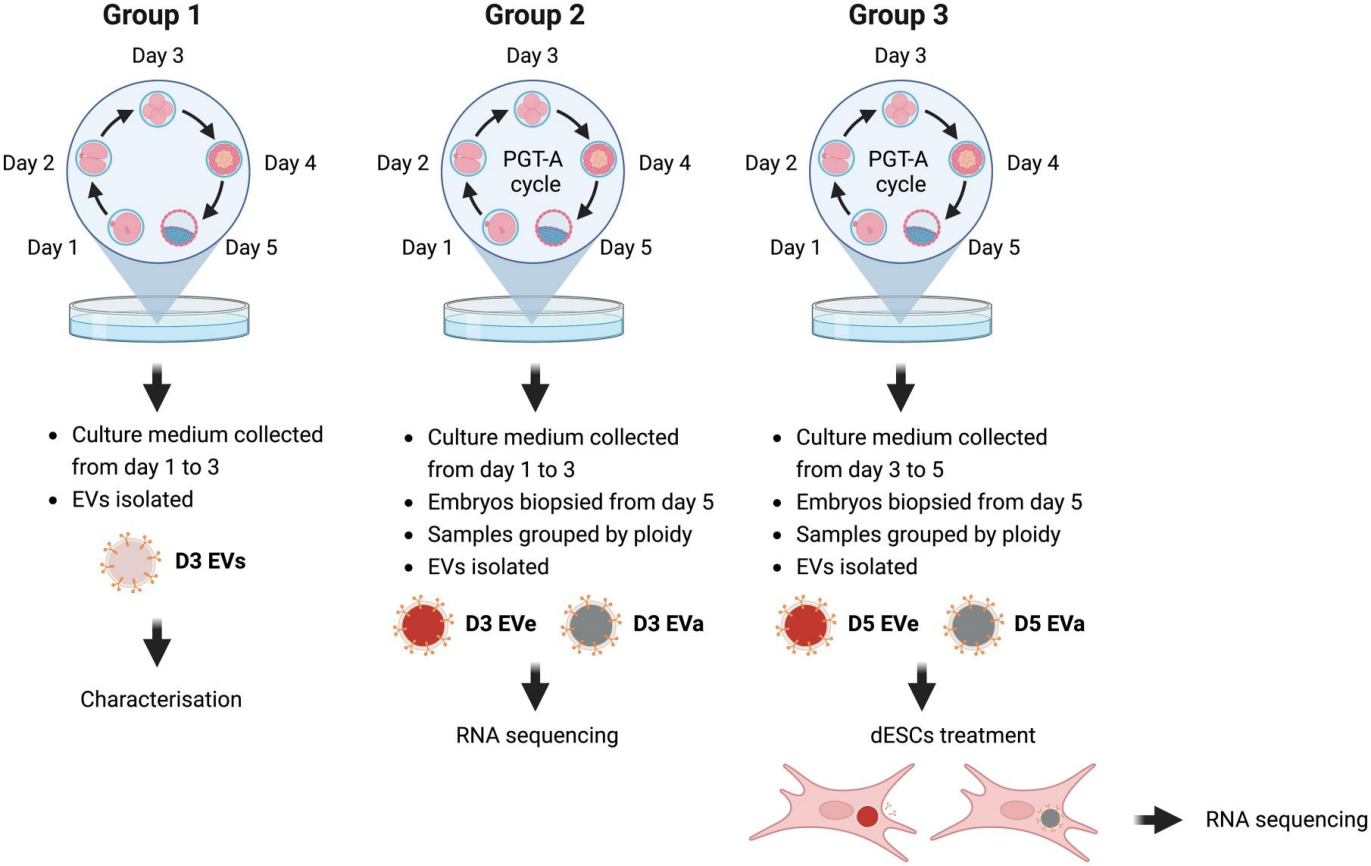
**Experimental design using human embryo-secreted extracellular vesicles isolated from embryo culture medium**. Human embryos created with IVF were cultured in sequential medium between Days 1 and 7 of development. According to this culture system, the embryos were transferred to new medium on Days 3 and 5 of development. The spent medium was collected on these days and EVs were isolated to define two versions of EVs: Day 3-EVs (D3 EVs) denoting the EVs isolated from medium where embryos were cultured between Days 1 and 3 of embryo development and Day 5-EVs (D5 EVs) denoting the EVs isolated from medium where embryos were cultured between Days 3 and 5 of development. We define three groups of EV samples depending on the purpose of experiments. In Group 1, D3 EVs were used for the experiments intended for the characterization of embryo-secreted EVs. Group 2 was used for the exploration of the diagnostic capacities of the RNA cargo in D3 EVs. In this group, all embryos were from PGT-A cycles and were diagnosed as euploid (D3 EVe) or aneuploid (D3 EVa). In Group 3, D5 EVs of diagnosed embryos (D5 EVe or D5 EVa) were used to treat dESCs. Graphic created with BioRender software. EV, extracellular vesicles; PGT-A, preimplantation genetic testing for aneuploidy; EVe, euploid EVs; EVa, aneuploid EVs; dESCs, decidualized endometrial stromal cells.

For the first experimental approach of the present study, we characterized the size and concentration, as well as the transcriptomic profile, of EVs derived from undiagnosed embryos grown in IVF culture media for 48 h (Fig. 1; Group 1). Day 3 EVs (D3 EVs) were derived from embryos cultured between Days 1 and 3 of development.

For the second step, aimed at identifying potential ploidy biomarkers, we have used a second cohort of samples (Fig. 1, Group 2) to compare the transcriptomic profile (EV-RNA) of D3 EVs derived from embryos diagnosed for ploidy at the blastocyst stage. Based on the PGT-A results, these EV-RNA were classified as euploid (D3 EVe) or aneuploid (D3 EVa).

In the third step, the last cohort of samples (Fig. 1, Group 3) included EVs derived from embryos grown between Days 3 and 5 of development (Day 5 EVs, D5 EVs), which were used to treat decidualized endometrial stromal cells (dESCs), to investigate the plausibility of a differential response of cells to the uptake of aneuploid or euploid embryo-exported EVs (EVa versus EVe).

### IVF procedures, trophectoderm biopsy, and PGT-A

All embryos were obtained after controlled ovarian stimulation that was performed using a GnRH-agonist or antagonist protocol. Oocyte retrieval occurred 36 h after triggering of ovulation and insemination was achieved with ICSI. Denudation of cumulus oophorous was performed by a brief exposure to HEPES-buffered medium (Quinn’s Advantage Medium with HEPES, Cooper Surgical, Trumbull, CT, USA) supplemented with human serum albumin and containing 20 IU/ml hyaluronidase solution (FUJIFILM Irvine Scientific, Santa Ana, CA, USA) and then, by removing the corona cells through denuding pipettes of decreasing diameter. After assessing their integrity, metaphase II oocytes were considered for injection. Injected oocytes were then rinsed and placed into pre-equilibrated sequential culture medium (Quinn’s Advantage Cleavage, Cooper Surgical) supplemented with Serum Substitute Supplement (SSS) (FUJIFILM Irvine Scientific) at 37°C in a controlled atmosphere (6% CO_2_ and 5% O_2_). Fertilization was assessed 17 h after insemination and a media change-over was performed (Quinn’s Advantage Cleavage, Cooper Surgical) supplemented with SSS.

After a media change-over on Day 3 post-insemination (Quinn’s Advantage Blastocyst, Cooper Surgical) supplemented with SSS, embryos were cultured until blastocyst stage, in a single drop of culture media under a controlled humidified atmosphere.

Day 5, 6, and 7 expanded blastocysts underwent trophectoderm biopsy. Analysis was performed in a reference genetic laboratory (Eurofins Genoma Group, Rome, Italy) as previously published (Fiorentino *et al.*, 2014). Immediately after biopsy, the blastocyst was vitrified.

### Collection of spent medium

**Figure 2.**
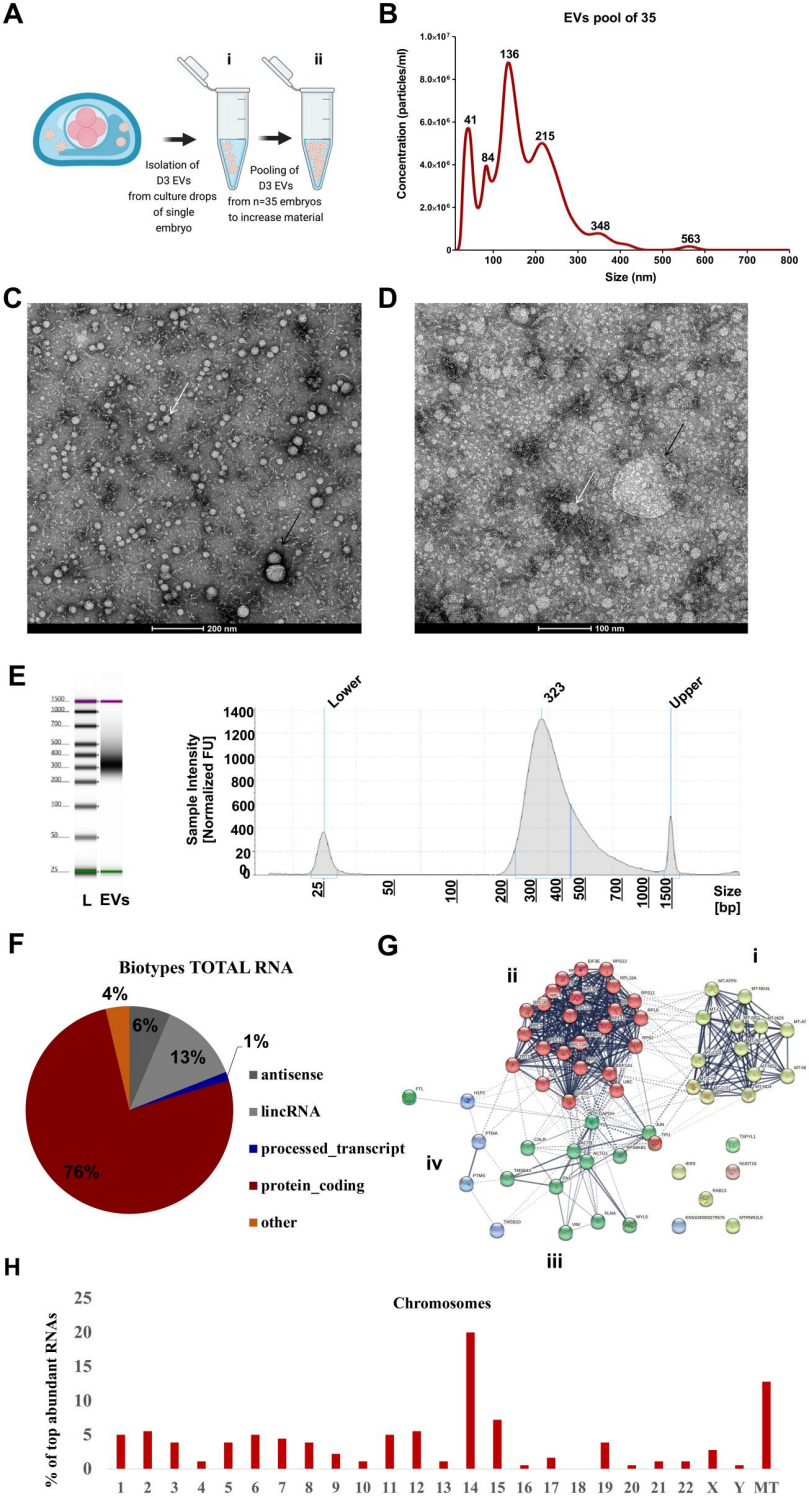
**Exploration of human embryo-secreted extracellular vesicle RNA cargo**. (**A**) Embryo-derived EVs were recovered from spent medium where single embryos were cultured for 48 h between Days 1 and 3 of development. Spent media samples from 35 single embryo cultures (i) were pooled to create a single pool (ii), which was used to isolate EVs (Group 1). (**B**) Representative NTA (nanoparticle tracking analysis) showing concentration (particles/ml) and size (nm) of embryo-derived EVs found in one pool sample (Aii). (**C**, **D**) TEM (transmission electron microscopy) showing EVs found in single embryo culture media (Ai). Black arrow indicates a larger EV and white arrow indicates a smaller EV. Scale bar is 200 nm (C) and 100 nm (D). (**E**) A representative Agilent 4200 TapeStation reading from a *Total RNA* library prepared using a pool of embryo EVs (Aii). (**F**) Biotype distribution across the RNAs present in EVs (n = 3 pools). (**G**) The 60 most abundant RNAs conserved across n = 3 pools of D3 EV and that code for proteins were used as an input in MCL (Markov cluster algorithm) analysis on STRING (protein–protein interaction networks functional enrichment analysis). An inflation parameter of 2 was used to identify four major clusters. The i (yellow) cluster consists of RNAs coding for mitochondrial proteins, the ii (red) RNA ribosomal processing and biogenesis, the iii (green) focal adhesion and intracellular trafficking, and the iv (blue) micromolecule biosynthesis. (**H**) The chromosomes most represented by the most abundant RNAs were 14 (20%) and mitochondria (MT; 12.7%). EV, extracellular vesicle; L, ladder; lincRNA, long intergenic non-coding RNAs.

As shown in Fig. 1, for Group 1 we collected spent culture medium (30 μl) from embryos (n = 105) grown in IVF culture media for 48 h between the stage of zygote and cleavage (D3), to investigate the size, concentration, and transcriptomic profile of D3 EVs. Media samples from n = 35 single cleavage embryos cultures were pooled to create a single pool for a total of n = 3 biological replicates (Fig. 2A). For Group 2, we collected spent culture medium from D3 embryos before they underwent biopsy later in development to compare the transcriptomic profile between euploid (n = 175) (n = 5 biological replicates) and aneuploid (n = 140) (n = 4 biological replicates) embryos. For Group 3, we collected spent culture medium from euploid (n = 187, 1 pool) and aneuploid (n = 142, 1 pool) expanded blastocysts on D5 with the aim of treating dESCs. Only spent culture medium from embryos and blastocysts without signs of degenerating cells during ART cycles were included. No selection based on patients’ characteristics was used for the collection of embryo spent medium and the collection was performed in order to have a maximum of two spent medium drops from a single patient. All samples were stored at −80 °C until use. Following biopsy at the blastocyst stage and PGT-A, the ploidy diagnosis was available for each embryo and corresponding spent medium sample. All samples from Group 2 and 3 were sub-grouped into a euploid sub-group or an aneuploid sub-group. Pools were used to increase the starting material and quality of downstream analyses. Spent media from embryos with mosaicisms and aneuploidies compatible with pregnancy (trisomy 13, 16, 18, 21, and 22) were excluded. Seventy-five percent of aneuploidies considered were complex abnormalities while the remaining were single trisomies or monosomies. For Group 2, media samples from n = 35 D3 embryos were pooled in order to obtain n = 5 euploid and n = 4 aneuploid pools. For Group 3, media samples from D5 were pooled to create an n = 1 euploid and n = 1 aneuploid pool used for cell treatment purposes.

### EV isolation

Embryo spent culture media were subjected to differential centrifugation to isolate EVs. Briefly, in order to remove cells, dead cells, and cellular debris, spent media of embryos/blastocysts were centrifuged at 300 × g for 10 min and at 2000 × g for 20 min; all centrifugation was performed at 4°C. The supernatants were transferred to 1.5 ml thick wall polycarbonate tubes (Beckman Coulter Inc., Carlsbad, CA, USA, item no. 357448) and an equal amount of PBS, filtered three times with a 0.1 µm filter, was added. Samples were ultra-centrifuged at 110 000 × g for 120 min at 4°C in a TLA55 rotor (Beckman Coulter Inc.) using an Optima TLX centrifuge (Beckman Coulter Inc.) to pellet EVs. The supernatants were removed completely and pellets were washed with 1 ml PBS and centrifuged for 60 min at 110 000 × g at 4°C.

For Group 1, pellets containing EVs from undiagnosed embryos (D3-EVs), and for Group 2, pellets containing euploid embryo-derived EVs (EVe) and aneuploid embryo-derived EVs (EVa), were used for RNAseq analysis. Pellets containing D5 EVe and D5 EVa (Group 3) were suspended in an adequate volume of PBS filtered three times with a 0.1-µm filter (50–100 µl) and the protein concentration was measured by Bradford assay and by NanoDrop8000, to assess EVs purification. We ensured that only samples with a protein content of at least 10 µg were utilized for the downstream experiments. All samples were stored at −80 °C until use. Fresh media supplemented with 10% SSS were subjected to the same procedures to ensure proper negative controls for each experiment.

In order to fully assess the size and the concentration of embryo-derived EVs and to visually confirm their shape and abundance, D3-EVs (Group 1) were pooled (n = 35) to create a single sample. This sample was used to isolate EVs as described previously (Giacomini *et al.*, 2017), to perform nanoparticle-tracking analysis (NTA) and transmission electron microscopy (TEM).

### Nanoparticle-tracking analysis

Particle size distribution and concentration of D3-EVs (Group 1) were determined by NTA, using a NanoSight LM10-HS instrument (NanoSight Ltd, Amesbury, UK). Briefly, the sample was diluted 1:40 with PBS, filtered three times with a 0.1-µm filter and concentrations adjusted, in order to specifically fit the optimal working range (20–50 particles per frame) of the instrument. Recording and video processing were performed as described previously (Giacomini *et al.*, 2017).

Results from all measurements were combined to obtain a particle size distribution and the total particle concentration corrected for the dilution factor. EVs from spent media of euploid and aneuploid Day 5 embryos were analyzed as single drops following the same procedure.

### Transmission electron microscopy

Preparation for TEM analysis of freshly purified D3 EVs (Group 1) was carried out using the method described by Giacomini *et al.* (2017) to visually confirm EVs shape and abundance.

### Endometrial stromal cell culture and decidualization

Human ESCs were isolated from healthy endometrial biopsies immediately after collection, according to Viganò *et al.* (1993). Briefly, endometrial tissue was gently minced into small pieces, and incubated for 2 h, at 37°C in a shaking water bath, in 10 ml RPMI supplemented with 0.1% collagenase (Sigma-Aldrich, St Louis, MO, USA). After the enzymatic digestion, digested tissue was filtered through nylon strainers of 40 μm pore size (BD Biosciences, Franklin Lakes, NJ, USA) and the flow-through was centrifuged at 500 x g for 5 min at room temperature and resuspended in 10 ml of complete medium, consisting of RPMI1640 (Lonza, Basel, Switzerland) supplemented with 10% foetal bovine serum (Sigma-Aldrich), 1% L-glutamine (Lonza), and 1% antibiotic/antimycotic (Lonza). Subsequently, stromal cells were separated from large clumps of epithelium by a 10-min period of differential sedimentation at unity gravity. The top 8 ml of medium, which predominantly contained stromal cells, were collected slowly and then centrifuged at 500 x g for 5 min; while the bottom 2 ml, which contained glands and epithelial cells were discarded. After centrifugation, the stromal-enriched fraction was suspended in 5 ml of complete medium, seeded in 24-well plates (Costar, Corning, NY, USA) and allowed to adhere selectively to culture plates for 20 min at 37°C, in a humidified atmosphere with 5% CO_2_ in air. Thereafter, non-attached epithelial cells still present were removed by changing media, in order to obtain a purified ESCs culture. ESCs were maintained in culture under standard conditions until 70–80% confluence. ESCs were decidualized (dESCs) by exposing them to 10 nM estradiol (E2) (Sigma-Aldrich), 1 μM progesterone (P4) (Sigma-Aldrich), and 0.5 mM 8-bromoadenosine-cyclic monophosphate (cAMP) (Sigma-Aldrich) twice every 48 h, up to 4 days (Yu *et al.*, 2016).

### ESC treatment with euploid and aneuploid embryo-derived EVs

After 72 h of decidualization, dESCs (n = 5) were either left untreated or treated for 24 h with euploid EVs (EVe) or aneuploid EVs (EVa). Specifically, 50 μg/ml EVe or EVa were added to culture medium supplemented with E2, P4, and cAMP. Non-decidualized ESCs were used as a control.

### RNA extraction from ESCs

ESCs and dESCs treated with or without EVs were washed with PBS and lysed with 350 μl of a solution containing RLT (Qiagen, Hilden, Germany) and β-mercaptoethanol (10 μl in 1 ml RLT; Sigma-Aldrich). The lysates were processed in order to perform RNA extraction, using the RNeasy Plus Micro Kit (#74034, Qiagen) according to the manufacturer’s instructions.

### RNA sequencing and analysis of endometrial cells

Libraries were prepared using the TruSeq Stranded mRNA kit (Illumina, San Diego, CA, USA) obtaining an average of 30 million 100 bp single end reads per sample. Sequencing was performed on NovaSeq 6000 platforms (Illumina). Reads were trimmed using Trimmomatic, version 0.32 (USADELLAB.org) to remove adapters and to exclude low-quality reads from the analysis. The remaining reads were then aligned to the reference genome hg38, Gencode version 31, using the STAR aligner v2.5.3a (Illumina). FeatureCounts (Bioconductor, Boston, MA, USA) was used to assign reads to the corresponding genes. Expressed genes were defined as those genes showing at least 1 count per million reads (cpm) on at least five different samples, where the number 5 is the size of each treatment group (undecidualized cells, decidualized cells, euploid EVs, aneuploidy EVs). According to this definition, 14580 expressed genes were identified. The top 5000 most variable genes were used to perform unsupervised hierarchical clustering, as implemented in the pheatmap R package, and principal component analysis (PCA), applying the prcomp R function to the preliminary centered and scaled data (R software, Indianapolis, IN, USA). Differential gene expression (DGE) analysis was performed using the DESeq2 Bioconductor library, version 1.22.1, with no assumption about prior distribution (option betaPrior=FALSE) and applying independent filtering to the result. One single model was fitted on the whole dataset, considering the sample group and the patient of origin as covariates. The only comparison that was performed separately was ‘Decidualized versus Undecidualized’, for which a paired model was fitted on the 10 considered samples only. Significance was defined either as a false discovery rate (FDR) < 0.05 or applying the SEQC cut-off (raw *P*-value < 0.01 and an absolute value of log2FoldChange greater than 1). Raw data were uploaded to GEO (accession number GSE234338).

### RNA sequencing of EVs and differential gene expression analysis

Libraries were prepared using the SMART-Seq Stranded Kit (Takara Bio, Shiga, Japan), using a single cell approach that circumvents RNA extraction. We obtained an average of 30 millions of 75 bp single-end reads per sample. Sequencing was performed on NextSeq 500 platform (Illumina). Reads were trimmed using Trimmomatic, version 0.32 (USADELLAB.org), to remove adapters and to exclude low-quality reads from the analysis. The remaining reads were then aligned to the reference genome hg38, Gencode version 31, using the STAR aligner v2.5.3a (Illumina). The counting procedure to assign mapped reads to the corresponding genes was performed using featureCounts (Bioconductor) and it was repeated many times, setting different parameters. The first time, only reads mapped on exonic regions were assigned to the corresponding genes; the second time all the reads mapped on transcripts were considered; and the third time all the reads aligned somewhere on gene bodies were included. Each of these procedures was repeated twice, performing reversely stranded or unstranded read counting. This was done because the library preparation kit was supposed to be reversely stranded, but from the MultiQC quality check (Infer Experiment statistic) we saw that percentages of reads matching the strandness of the overlapping transcripts are equally distributed across the two strands. Moreover, owing to the large fraction of intronic sequences present in the libraries (65.5% introns, 4.8% exons), the counting performed on the full gene bodies was preferred as it was able to include a higher number of reads. All the following analyses were performed considering only the matrices obtained by counting on transcripts and on genes. Only ‘expressed’ genes were reported. As ‘expressed’ were defined genes with at least 1 cpm on at least four different samples, where the number 4 is the size of the smallest group of samples (aneuploid). DGE analysis was performed using the DESeq2 Bioconductor library (Bioconductor), version 1.26, with no assumption about prior distribution (option betaPrior=FALSE) and applying independent filtering to the result. The comparison was defined as ‘Aneuploid versus Euploid’, using the euploid embryo-derived EVs transcriptome profile as reference. Significance was defined either as FDR < 0.05 or applying the SEQC cut-off (raw *P*-value < .01 and an absolute value of log2FoldChange greater than 1). Raw data were uploaded to GEO (accession number GSE234338).

### Correlation of EV RNA sequencing with embryo single cell sequencing data

The expression profiles of embryo-derived EVs samples (n = 3) were compared to those retrieved from the publicly available datasets of Petropoulos *et al.*, (2016) (ArrayExpress E-MTAB-3929) and Yan *et al.*, (2013) (GEO accession number GSE36552).

Both these studies were conducted using single-cell RNA sequencing (scRNAseq): cells from human embryos at different developmental stages were isolated and their transcriptomes were sequenced and analyzed. The first step needed to compare these data with our EVs samples was to reshape the scRNAseq data into a ‘pseudo-bulk’ format. To do so, the expression values of all the cells obtained from the same embryo were aggregated, calculating the overall expression for that embryo.

Then, common genes detected in both our samples and in the considered dataset were identified and further analyses were conducted on this list of shared genes only.

Expression values from different studies were also harmonized by applying the *removeBatchEffect* function, implemented in the *limma* Bioconductor library (Bioconductor), for a better comparison and diminishing the effect of technical biases. Finally, ‘pseudo-bulk’ batch-regressed data were compared with the transcriptomic profiles of EVs samples by performing a PCA, unsupervised clustering and correlation analysis.

### Functional enrichment analysis

Functional enrichment analysis was performed using various tools including EnrichR, WebGestalt, STRING and g: Profiler to identify the Pathways and Gene Ontology (GO: Biological Processes, Molecular Function and Cellular Component) enriched in the various DEG datasets. Herein we used STRING normal geneset-based ranked analysis to explore whether the DEGs are involved in known and predicted protein–protein interactions (Szklarczyk *et al.*, 2015). The Markov clustering algorithm is a fast and scalable unsupervised cluster algorithm for networks used to reveal clusters of proteins that are regulated together. The g: Profiler tailor-made algorithm g: SCS was used to correct the results for multiple testing (Raudvere *et al.*, 2019). WebGestalt was used to perform the gene set enrichment analysis (GSEA).

## Results

### RNA characterization of EVs derived from Day 3 embryos

For the first experimental approach of the present study, we isolated EVs from culture media where embryos developed for 48 h from the stage of zygote to cleavage (D3 EVs; Group 1). D3 EVs from 35 embryos were pooled to create a single D3 EV pool for transcriptomic analysis (Fig. 2A). NTA revealed that EVs had on average a size of 191.1 ± 17.61 nm (mean diameters ± SD) and a concentration of 2.62 × 10^10^ ± 6.03 × 10^9^ particles/ml (Fig. 2B). TEM confirmed their shape and abundance (Fig. 2C and D).

#### Findings from RNA sequencing

To study the transcriptomic profile of these undiagnosed D3 EVs, we used a commercial kit widely applied in single-cell transcriptomics that uses a random-priming approach to capture the total transcriptome. Hence, instead of analyzing only mRNA fragments, we sought to analyze total RNA, which would increase our chances to identify credible biomarkers. Since we did not perform RNase treatment on EV samples before RNA sequencing, we cannot exclude that the analysis also included RNA species on the outer surface of EVs (Hill *et al.*, 2013). Moreover, we opted to utilize only D3 EVs and not D5 EVs, as biomarkers earlier in development would be more practical in an infertility treatment timeline. In order to analyze the RNA in EVs, we constructed a cDNA library representing the total RNA fragment landscape (Fig. 2E), which we sequenced to find that the 76% of the RNA fragments detected in embryonic EVs come from embryo cell transcripts that code protein while 13% are of long non-coding RNA (lincRNA) origin (Fig. 2F). We performed the experiment on three independent pools, each composed of 35 samples of D3 EVs, and identified 179 RNAs that were common (intersection) among the top 1000 RNAs in each of the three datasets (Supplementary Table S1). These were mainly RNAs involved in ribosomal biogenesis, focal adhesion, intracellular trafficking, and macromolecule biosynthesis, as well as RNAs coding for mitochondrial proteins (Fig. 2G). Interestingly, the most abundant RNAs contained in EVs originated predominantly from genes on the chromosome 14 (20%) and mitochondria (12.7%) of embryos (Fig. 2H). The transcriptomic profile of the EVs isolated from control culture media did result in a negligible level of transcripts (data not shown). The output of biological pathways (BP) and cellular components (CC) analyses using as an input the identified 179 EV-RNAs (Supplementary Table S2) was similar to the respective output of BP and CC in a recent study that used for input whole embryo-RNA sequencing data (Groff *et al.*, 2019). This suggested that the origin of the RNA sample was indeed embryonic.

#### Correlation of RNA sequencing data from D3 EVs and embryonic cells

**Figure 3.**
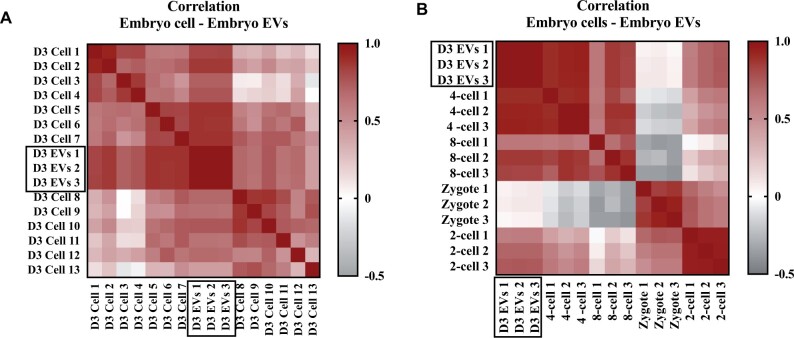
**Correlation of expression matrix between human embryo extracellular vesicle RNA and embryo cell-RNA**. Single-cell RNA sequencing raw data on embryos was available in literature from Petropoulos *et al.* (2016) (**A**) and Yan *et al.* (2013) (**B**) and was used to examine correlation with RNA sequencing data from embryo-derived D3 EVs in our study to validate the hypothesis that EVs are proxy of the tissue of origin. A: Petropoulos *et al.* (2016) performed RNA sequencing on 13 single cells from embryos desegregated on Day 3 of development. The correlation of the expression matrix shows strong correlation between RNA expression in Day 3 (D3) embryo cells and RNA fragments identified in EVs of Day 3 embryos (n = 3). B: Yan *et al.* (2013) performed RNA sequencing on zygotes (n = 3) and single cells from different stages of embryo development: 2-cell (n = 3), 4-cell (n = 3) and 8-cell (n = 3). The correlation of the expression matrix shows strong correlation of RNA expression in 4- and 8-cell embryos with RNA fragments in D3 EVs. EV, extracellular vesicle.

We then sought to find more evidence that the D3 EV transcriptomic profile was a proxy of blastomere transcriptome. We looked at the correlation of our RNA sequencing results with the RNA sequencing results of two studies that utilized a similar methodological approach (single-cell sequencing) on embryo cells of similar developmental stage (Fig. 3A and B). We found a strong correlation of our Day 3 embryo EV-RNA sequencing data with that of Petropoulos *et al* (2016) and with 4-cell and 8-cell stage cleavage embryos of Yan *et al* (2013). For the Petropoulos correlation (Fig. 3A) the *r* values ranged from 0.69 to 0.88 and only for one cell it was 0.43. For the Yan correlation (Fig. 3B), the correlation of Day 3 EVs with cells from 4-cell embryo yielded r values between 0.86 and 0.94, with cells from 2-cell embryo between 0.58 and 0.75, with cells from 8-cell embryo between 0.62 and 0.86 and with zygote the correlation was only 0.03 to 0.09. This could suggest that embryos secrete EVs predominantly at the cleavage stage with ≥4 cells. The similarity of EV-RNA profile in our study and the embryo cell-RNA profiles in the aforementioned studies reassured us that our methodology for RNA sequencing of embryo-derived EVs is credible.

### EVs derived from Day 3 embryos transcriptomic profile could serve for the development of ploidy biomarkers

#### Findings from RNA sequencing

**Figure 4.**
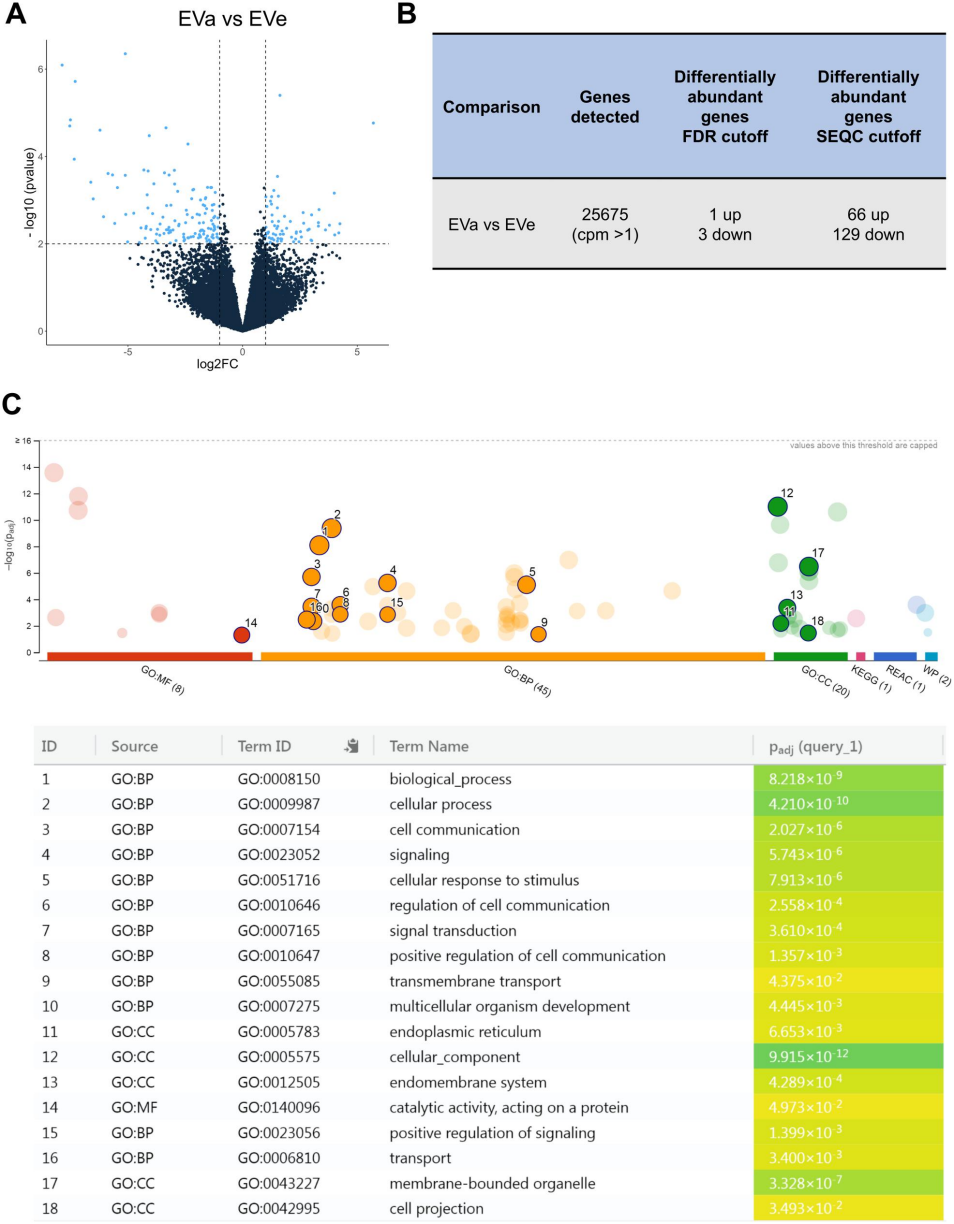
**Differential abundance in extracellular vesicle RNA cargo between euploid and aneuploid Day 3 embryos. (A**, **B**) Differential gene expression analysis with SEQC cut-off (sequencing quality control raw *P*-value < 0.01, fold change: 1) showing the genes differentially abundant between D3 EVs collected from aneuploid embryos (D3 EVa; n = 4 biological replicates) and the ones collected from euploid embryos (D3 EVe; n = 5 biological replicates). A: Volcano plot. B: The comparison was defined as ‘EVa vs. EVe’, using the euploid embryos-derived EVs as reference. The number of genes differentially abundant when implementing two different cut-offs is indicated. (**C**) Enrichment overrepresentation analysis results of D3 EVa and D3 EVe transcriptomes: GOSt multiquery plot of Manhattan. All differentially abundant genes were selected and tested against BP, CC, Reactome, and Wikipathway collections. The most significant results (FDR < 0.05) are highlighted. EV, extracellular vesicle; BP, biological process; CC, cellular component; FDR,false discovery rate; GOSt, gene ontology statistics.

For the second step, we sequenced the total RNA of D3 EVs secreted from embryos (Group 2) diagnosed as euploid (EVe) or aneuploid (EVa) when subsequently biopsied and analyzed at the blastocyst stage. Nine pools of EVs derived from euploid (EVe n = 5) or aneuploid (EVa n = 4) embryo were used. RNA sequencing revealed the following numbers of expressed genes: Counting on transcripts—reversely stranded: 29 326 expressed genes, counting on transcripts—unstranded: 26 545 expressed genes, counting on genes—reversely stranded: 29 055 expressed genes, counting on genes—unstranded: 25 675 expressed genes. Considering the model ‘counting on genes—unstranded’, RNA sequencing revealed that aneuploid embryos secreted EVs that were enriched in 66 RNA fragments, while 129 RNA fragments were less abundant in aneuploid EVs (EVa) compared to euploid EVs (EVe), considering the SEQC cut-off (raw *P*-value < 0.01 and a logFC > 1 or logFC < −1) (Fig. 4A and B and Supplementary Table S3).

#### Functional enrichment analysis

The differentially ‘expressed’ gene transcripts from the comparison between EVa and EVe were used as input for functional enrichment analysis. As shown in Fig. 4C, GO analysis identified statistically significant genes (FDR < 0.05) that were associated with BP such as *cell communication*, *signaling*, *cellular response to stimulus*, *transmembrane transport*, *multicellular organism development, and developmental process* (orange dots GO: BP; Fig. 4C). Additionally, EVas were found to be enriched with transcripts of genes related to *endoplasmic reticulum*, *endomembrane system*, *membrane-bounded organelle*, and *cell projection* (green dots; GO: CC; Fig. 4C).

Furthermore, EnrichR was used to analyze the up- and down-regulated terms in EVa (Supplementary Table S4). Among the more abundant RNAs, EVa exhibited a statistically significant enrichment of transcripts of genes related to BPs including *Vesicle Budding From Membrane, COPI Coating Of Golgi Vesicle, COPI-coated Vesicle Budding, Golgi Transport Vesicle Coating, positive regulation of apoptotic signaling pathway*, and *apoptotic process* (*P*-value < 0.05). Conversely, transcripts of genes related to *Filopodium Assembly, Negative Regulation of Stem Cell Population Maintenance, Uridine Transport, and Positive Regulation of Toll-Like Receptor 2 Signaling Pathway*, showed a statistically significant downregulation in EVa (*P*-value < 0.05), among others. Moreover, EVa also displayed a statistically significant enrichment of transcripts of genes related to CCs relevant to vesicle transport.

**Figure 5.**
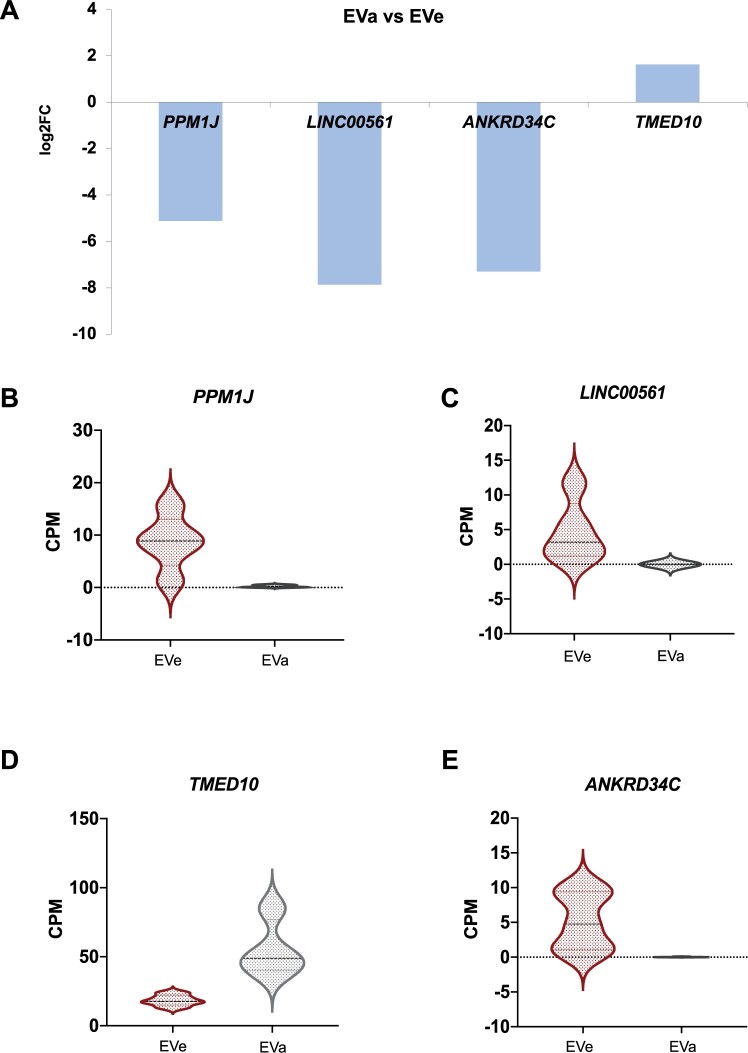
**Differentially abundant RNAs between aneuploid and euploid extracellular vesicles**. According to differential gene expression analysis, five RNA species showed differential abundance (FDR: *P* adjusted < 0.05) in EVa (**A**). In lower abundance: PPM1J (**B**; FDR = 0.010), LINC00561 (**C**; FDR = 0.010), and ANKRD34C (**D**; FDR = 0.016) while in higher abundance: TMED10 (**E**; FDR = 0.025). CMP, counts per million; FDR, false discovery rate; PPM1J, protein phosphatase, Mg^2+^/Mn^2+^-dependent 1J; LINC00561, long intergenic non-protein coding RNA 561; ANKRD34C, ankyrin repeat domain 34C; TMED10, transmembrane p24 trafficking protein 10; EVa, aneuploid extracellular vesicles; EVe, euploid extracellular vesicles.

A more stringent analysis (FDR < 0.05) on RNA sequencing of EVs secreted from aneuploid embryos allowed for identification of four candidate RNAs that could serve as EV biomarkers of embryo aneuploidy (Fig. 5): *PPM1J (protein phosphatase, Mg2+/Mn2+ dependent 1J), LINC00561 (long intergenic non-protein coding RNA 561), ANKRD34C (ankyrin repeat domain 34C)*, and *TMED10 (transmembrane p24 trafficking protein 10)* (Fig. 5A). Out of the four RNAs, TMED10 (Fig. 5D) was the only RNA enriched in the EVs secreted from aneuploid embryos.

### EVs derived from Day 5 embryos induced upregulation of MUC1 gene in dESCs

Finally, we wanted to explore the notion that aneuploid embryo-derived EVs (EVa) could regulate the transcription in the endometrium differently compared to euploid EVs (EVe). For this aim, we used D5 EVs (Group 3) rather than D3 EVs (Group 2), based on the gold-standard assumption that *in vivo* embryo implantation occurs on Day 5 of development.

#### Findings from NTA

**Figure 6.**
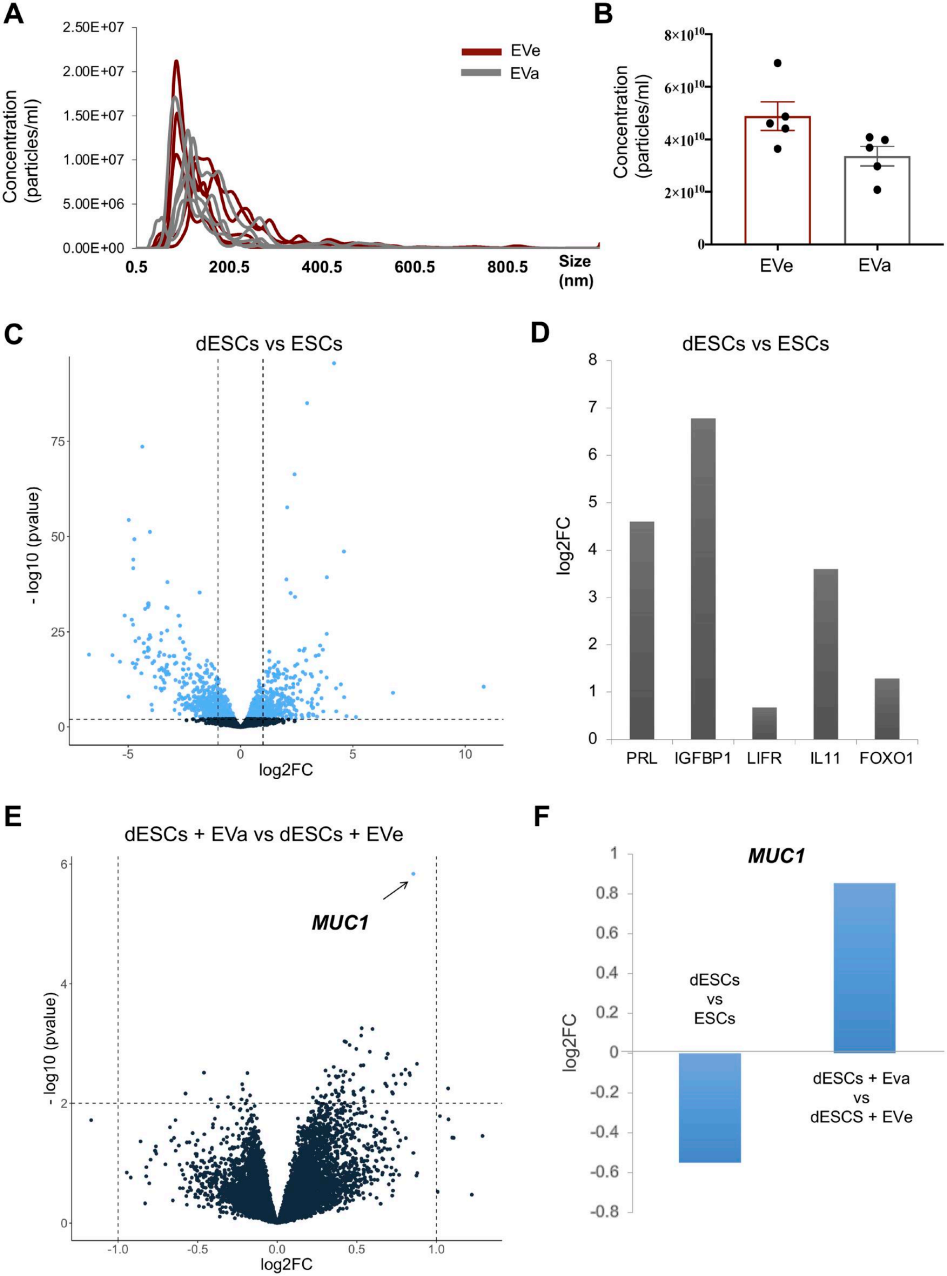
**The response of decidualized endometrial cells to the uptake of extracellular vesicles secreted from Day 5 euploid and aneuploid embryos. (A**, **B**) Individual EVe and EVa samples (not pools) were analyzed with ΝTA. A: NTA showing concentration (particles/ml) and size (nm) of D5 EVe (n = 5, red line) and D5 EVa (n = 5, grey line) embryos. B: Quantification of the NTA results; EVe mean concentration: 48868407976 ± 5443394193 particles/ml and EVa mean concentration: 33595295619 ± 3743527687 particles/ml (p = ns, fc = 1.45), ± values denote SEM. (**C**, **D**) Successful decidualization of ESCs is confirmed by DGE analysis between dESCs and ESCs. C: Volcano plot showing that decidualization of ESCs caused a remarkable transcriptomic response characterized by the upregulation of 1023 and downregulation of 1111 genes (n = 5 patients, FDR <0.05). D: Known markers of decidualization were upregulated in cells treated with the decidualization protocol: PRL (prolactin; FDR < 0.0001), IGFBP1 (insulin-like growth factor-binding protein 1; FDR < 0.0001), LIFR (leukemia inhibitory factor receptor; FDR = 0.02), IL11 (FDR < 0.0001), FOXO1 (forkhead box protein O1; FDR = 0.001). (**E, F**) DEG comparing dESCs treated with D5 EVe and D5 EVa (50 μg/ml; 24 h; n = 5 patients, cut-off set FDR < 0.05). E: Volcano plot showing MUC1(mucin 1) as the only gene upregulated in dESCs treated with D5 EVa (dot shown with arrow). F: MUC1 gene differential expression in the following comparisons: ESCs versus dESCs (log2FC = −0.54, FDR = 0.0028) and dESCs treated with D5 EVe versus dESCs treated with D5 EVa (log2FC = 0.85, FDR = 0.0201). MUC1 was downregulated in dESCs (when compared to ESCs) and upregulated in dESCs treated with EVa (when compared to EVe). EV, extracellular vesicle; FDR, false discovery rate; EVe, euploid EVs; EVa, aneuploid EVs; NTA, nanoparticle tracking analysis; DGE, differential gene expression; ESCs, endometrial stromal cells not decidualized; dESCs, decidualized endometrial stromal cells.

NTA measurements demonstrated that embryo-derived D5 EVa and EVe show similar size distribution profiles, with a diameter of 156.6 ± 52.87 and 148.1 ± 32.42 nm (mean ± SD), respectively (Fig. 6A). Moreover, it also suggested no significant difference in the number of EVs in spent media from euploid embryos compared to those present in spent media from aneuploid embryos. Specifically, mean concentration was 4.89 × 10^10^ ± 1.22 × 10^10^ particles/ml in EVe samples and 3.54 × 10^10^ ± 1.1 × 10^10^ particles/ml in EVa samples (Fig. 6B).

#### Treatment of dESCs with D5 EVs

Treatment of cells with the hormonal cocktail to mimic the decidualization-associated changes, induced a remarkable transcriptomic response characterized by the upregulation of 1023 and downregulation of 1111 genes (Fig. 6C). In line with this finding, well-established transcripts associated with decidualization (Makieva *et al.*, 2018) were upregulated in dESCs: *PRL (prolactin), IGFBP1 (insulin-like growth factor binding protein 1), LIFR (leukemia inhibitory factor receptor), and IL11, FOXO1 (forkhead box protein O1)* (Fig. 6D). Afterwards, we treated dESCs with D5 EVe or D5 EVa (50 μg/ml) for 24 h. When dESCs were treated with EVs secreted from aneuploid embryos, an increase in mucin 1 (*MUC1)* transcript was observed (Fig. 6E and F). Interestingly, *MUC1* was downregulated with decidualization (dESCs versus ESCs cells; Fig. 6F), consistent with the literature suggesting that *MUC1*, a high-molecular-weight mucin O-linked glyco-protein, is a physical barrier to receptivity for uterine adhesion in several species, including humans (Meseguer *et al.*, 1998).

#### GSEA

**Figure 7.**
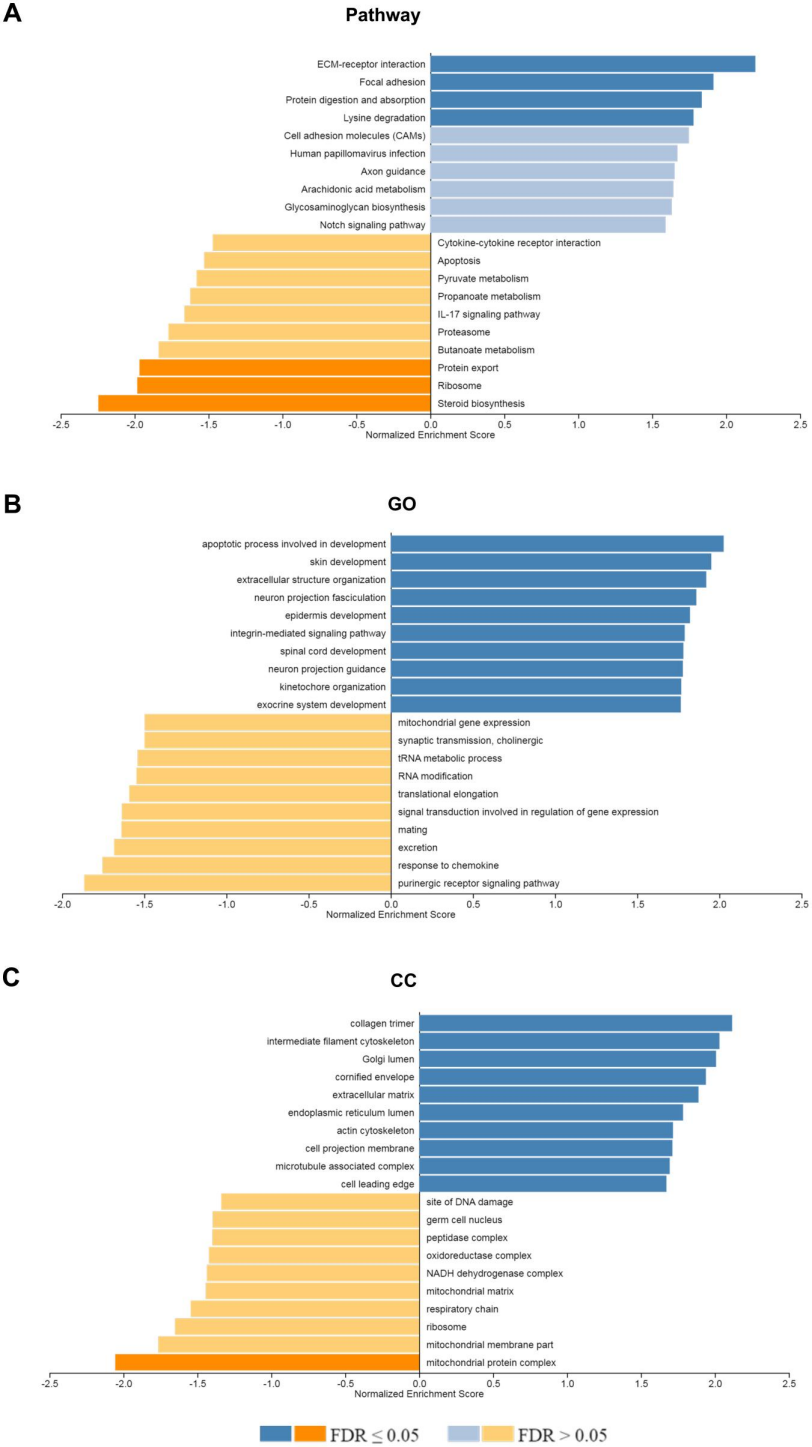
**Gene set enrichment analysis of gene expression comparison of decidualized endometrial stromal cells treated with Day 5 extracellular vesicles secreted from aneuploid or euploid embryos**. All the expressed genes were sorted based on their log2FC value for comparison between dESCs + EVa and dESCs + EVe, resulting in a pre-ranked gene list that was further used in a GSEA gene ontology (GO), cell component (CC), or pathway analysis. Only categories with an FDR < 0.05 and a minimum of 20 genes are presented. The NES (normalized enrichment score) of top enriched (blue bars) and top depleted (orange bars) terms in dESCs + EVa compared to dESCs + EVe are listed, categorized by GO, CC, or pathway annotations. WEB-based GEne SeT AnaLysis Toolkit (WebGestalt, http://www.webgestalt.org/) was utilized to perform the GSEA. dESCs, decidualized endometrial stromal cells; EVa, euploid EVs; EVa, aneuploid EVs; GSEA, gene set enrichment analysis; FDR, false discovery rate.

Following a 24-h exposure to EVa, dESCs exhibited a statistically significant enrichment in transcripts of genes related to *ECM-receptor interaction*, *focal adhesion*, *protein digestion, and absorption pathway* (FDR ≤ 0.05), as well as *cell adhesion molecules*, *arachidonic acid metabolism*, and *Notch signaling pathway* (FDR > 0.05; Fig. 7) when compared to dESCs exposed to EVe. Conversely, pathways such as *protein export*, *ribosome*, and *steroid biosynthesis pathway* (FDR ≤ 0.05), together with *IL-17 signaling pathway*, *apoptosis*, and *cytokine-cytokine receptor interaction* (FDR > 0.05) were decreased in dESCs treated with EVa. Additionally, GO terms analysis revealed that the transcriptomic profile of dESCs treated with EVa was associated with processes such as *apoptotic process involved in development*, *extracellular structure organization*, and *integrin-mediated signaling pathway* (FDR ≤ 0.05).

## Discussion

This is the first study describing the transcriptomic profile of EVs released by human aneuploid and euploid embryos and revealing a potential role of aneuploid embryo-derived EVs in limiting embryo implantation into the decidualized endometrium. Indeed, our results highlight a significantly different transcript abundance in EVs secreted by cleavage stage embryos diagnosed as aneuploid when compared to euploid. Moreover, dESCs respond to the uptake of aneuploid EVs by upregulating the expression of MUC1, implying a regulatory role of embryo EVs in conveying implantation signals to the endometrium.

Beyond their interesting feature as mediators of signals for the establishment of pregnancy, the potential of EVs to serve as preimplantation biomarkers of embryo competence is very attractive. Recently, differences in the miRNA cargo of embryo-derived EVs between good- and poor-quality embryos has been implicated in the establishment of a successful pregnancy (Poh *et al.*, 2023). Besides RNA, DNA present in EVs isolated from embryo culture medium has been utilized for non-invasive assessment of embryo chromosomal abnormalities by microarray-based comparative genomic hybridization assay (Veraguas *et al.*, 2021).

In the present study, we provide novel information on EVs isolated from human preimplantation embryo cultures, expanding the knowledge on their transcriptomic profile and function upon uptake by the human endometrial cells. The first major finding in our study is the demonstration that the EV transcriptomic profile from Day 3 embryos is indeed a proxy of the blastomere transcriptome. To establish the embryonic origin of EVs recovered in culture media, we used the data from two studies that applied single-cell RNA sequencing on human embryonic cells of similar developmental stage (Yan *et al.*, 2013; Petropoulos *et al.*, 2016). We found a strong correlation between our Day 3 embryo EV-RNA sequencing data with the RNA sequencing data deposited in the aforementioned studies for cleavage stage embryos: a result that reassured us that our methodology for EV-RNA sequencing is credible and that the EVs we derive are secreted by the embryos.

Thus, encouraged by the results obtained, we proceeded to investigating the abundance and transcriptomic profile of EVs derived from aneuploid compared to euploid embryos. Transcripts enriched in EVs from aneuploid embryos comprised genes involved in regulation of cellular communication, transmembrane transport but also cell signaling. Among them, the *TMED10* transcript, encoding a protein localized to the plasma membrane and Golgi cisternae of unclear function, was one of the most abundant. Recently, Zhang *et al.* (2020) provided ground-breaking evidence that many cytosolic proteins lacking secretion signal sequences rely on the TMED10 protein that oligomerizes to channel the cargoes into secretory vesicles. It is therefore plausible that aneuploid embryos express more TMED10 protein to increase the trafficking demand of informative cargoes from the embryo to the endometrium, possibly facilitated via EVs. Thus, besides being a promising transcript for the development of a non-invasive biomarker of embryo aneuploidy, *TMED10* enrichment in EVs may reveal a novel mechanism by which aneuploid embryos communicate their incompetence to the endometrial compartment. This remains, however, a speculation.

Support toward the presence of a different transcriptomic profile in EVs from aneuploid compared to euploid embryos is derived from the assessment of transcriptomic response of dESCs to the incorporation of these cargos, further along in our study.

RNA exchange between embryo and endometrium facilitated by EVs has been documented recently using a non-contact co-culture system showing transfer of labeled EV-RNA from trophoblasts to endometrium (Es-Haghi *et al.*, 2019). In a later study, the same group confirmed that embryo-derived EVs can induce significant transcriptomic alterations in the endometrial cells as opposed to non-embryo-derived EVs (Godakumara *et al.*, 2021).

In the present study, by treating dESCs with EVs released from aneuploid or euploid embryos, we have established a set of endometrial genes that is regulated in relation to the embryo ploidy. The signature resulting from incorporation of EVs derived from aneuploid embryos referred to genes relevant to extracellular matrix–receptor interaction and extracellular matrix structure organization. For euploid embryo-derived EVs, the response was associated with the upregulation of transcripts related to cytokine–cytokine receptor interaction, the IL-17 signaling pathway and steroid biosynthesis in dESCs. These results, which represent the second major finding of our study, support the involvement of intricate cytokine pathways (IL-17, IL-10, and tumor necrosis factor) in governing selective immune regulation during the successful embryo–endometrium dialogue (Simón *et al.*, 1998). Another interesting result derives from the DGE analysis of dESCs treated with aneuploid embryo-secreted EVs, showing an upregulation of *MUC1* expression (Meseguer *et al.*, 1998). MUC1 is a highly glycosylated glycoprotein present on the endometrial cell surface and decidual cells (Meseguer *et al.*, 1998; Chauhan *et al.*, 2015) and known to be involved in human endometrial receptivity since 1998. MUC1 is an extremely potent inhibitor of embryo attachment to the uterine epithelia in humans. *In vitro* implantation models indicate that MUC1 is lost only at the site of embryo attachment (Chauhan *et al.*, 2015), suggesting that factors expressed on the blastocyst surface or released from blastocyst trigger the loss of MUC1 at the point of contact. Removal of MUC1 permits conceptus attachment to the uterine lining through the expression of integrins and numerous possible adhesive molecules on the embryo and uterine surface. Therefore, the strong upregulation of MUC1 expression by dESCs in response to aneuploid embryo-derived EV uptake highlights a plausible crucial role of EVs in regulating optimal MUC1 expression in order to control implantation.

Some limitations of the present study deserve consideration. Embryo-derived EVs used to treat dESCs were pooled from a number of media drops. This procedure was required in order to increase the quantity of EVs for *in vitro* handling, detection, and experimentation. The *in viv*o conditions are difficult to reproduce but, in the virtual uterine cavity, the cell-to-cell communication is likely to be far more efficient. Furthermore, although *MUC1* has been previously shown to be expressed by decidual stromal cells (Chauhan *et al.*, 2015), we did not evaluate the transcriptomic effect of embryo-derived EV incorporation by the epithelial cells that are probably the main barrier to embryo implantation. Owing to the limited amount of the starting material, characterization of the embryo-derived EVs was not extensively documented here. Notably in this regard, the nature and the embryonic origin of the vesicles had been characterized in our previous study by western blot analysis and flow cytometry for specific markers (Giacomini *et al.*, 2017). In a previous study (Giacomini *et al.*, 2021), we demonstrated that: embryo-derived EVs were CD63+, CD9+, and ALIX+, as shown by western blotting; EVs isolated from embryos at different developmental stage were visualized by TEM after immunogold labeling with anti-CD63 and anti-CD9 antibodies; and the embryonic origin of embryo-derived EVs was confirmed by the presence of stemness gene transcripts and their enrichment in HLA-G protein. Of note, NTA measurements demonstrated that embryo-derived D5 EVa and EVe showed similar size distribution profiles. Finally, we did not perform RNase treatment on EV samples before RNA sequencing and we cannot exclude that our results may also derive from RNA species on the outer surface of EVs.

In conclusion, our study provides a unique insight into the transcriptomic landscape of EVs derived from euploid and aneuploid human embryos created *in vitro* following ICSI. We identify four transcripts that may be considered for the development of non-invasive PGT-A diagnostics based on the analysis of EVs recovered from spent culture medium. Finally, we propose a novel hypothesis whereby embryos disclose their aneuploidy via delivery of an EV cargo to the endometrium, which in turn upregulates MUC1 to reject implantation. We call for further research to consolidate this mechanism to advance our knowledge of implantation failure.

## Supplementary Material

hoae014_Supplementary_Data

## Data Availability

The data underlying this article will be shared on reasonable request to the corresponding author. The OMICS data is available on GEO (accession number GSE234338).
